# 

**Published:** 1885-02-20

**Authors:** 


### EDITORIAL NOTICES.

To the Profession.—Reports of operations upon the gall-bladder and ducts, whether previously published or not, are desired for a contribution to an important work, in which due credit will be given for any available matter; and all facts will be thankfully received by J. McF. Gaston, M. D., Atlanta, Georgia.

Death of Dr. Gaillard.—We are sorry to learn that Dr. E. S. Gaillard, of New York, the able editor of the Medical journal known by his name, died on Monday, the 2d day of February, instant.

Commencement Exercises.—The Southern Medical College Commencement exercises will take place at DeGive's Opera House, Atlanta, on the evening of March 4, 1885. The annual address will be delivered by the Rev. Mr. Armstrong, of this city. The occasion will, doubtless, be an interesting one, and a large audience will, in all probability, be present.

The Meeting of the American Medical Association at New Orleans, on the 28th of April next, during the existence of the great Exposition, will, probably, be the largest and most interesting ever held since its organization.

See the following new advertisements in this issue: Cocaine, by Parke, Davis & Co.; Crab Orchard Water; Medical College of Virginia; Papine; and Problems of Nature.

Snake Bites.—It is reported that there were 19,519 deaths in British India from snake bites in the year 1882, and that this is probably short of the average yearly number.

The Problems of Nature is the name of a scientific journal published in New York. The editor is the author of many bold and novel theories touching the great problems in science. Many of his thoughts are not only interesting, but startling in their originality.

The paper is well calculated to attract the attention of investigating minds. We invite special attention to the advertisement of the paper as found in this journal.

### *RECEIPTED.*

1883.—Drs. I. N. Bulloch, E. L. Mauck, B. B. Graham, L. N. Bailey, Robt. T. Smith, James Buchanan, Samuel Willis.

1884.—Drs. J. S. Miller, G. E. Flower, W. H. Stewart, N. B. Warren, E. W. Lane, J. D. Harrell, R. H. Edwards, C. A. Jordan.

1885.—Drs. R. E. Toombs, E. H. Wright, J. E. Rogers, N. C. Pyles, H. J. Sanders, J. W. Pettis, T. S. Parham, E. H. M. Parham, T. J. Hendley, L. S. Brownlee, W. H. Boykin, L. Allison, Jno. Gerdine, W. M. Coleman, P. C. Sircuit, Jno. Thomas, C. B. Thomas, J. W. V. Plummer.

